# Notes and Notices

**Published:** 1883-03

**Authors:** 


### NOTES AND NOTICES.

Homœopathy Ahead in San Diego, Cal.—The City Trustees met yesterday and reorganized the Board of Health. Dr. G. W. Barnes, who had previously been elected Health Officer, was chosen President, and Arnold Schneider, Secretary.

The Minneapolis Homœopathic Hospital has been opened for reception of patients. The movement for this purpose was started in 1881, and has, after overcoming many difficulties, now commenced its work. Stick to Hahnemann, brethren, and success is assured. Medical Board: D. H. Goodwin, M. D., Chairman; O. M. Humphrey, M. D.; W. H. Leonard, M. D.; S. M. Spaulding, M. D.; Adele S. Hutchison, M. D., and Wm. E. Leonard, M. D, Superintendent.

Alumni Association of the New York Homœopathic Medical College.—It is desired to organize this Association next Commencement Day. It is a good move and we wish it success.

Seventh Annual Meeting of the Homœopathic Ophthalmological and Otological Society.—The seventh annual meeting of the American Ophthalmological and Otological Society will be held at Niagara Falls in June. The President of the Society is especially desirous that the meeting be an interesting and profitable one. To that end he hopes that a large number of brief but practical papers may be presented, embodying, as far as may be, the clinical experience of the members. The meeting will be held on the day previous to that appointed for the opening of the American Institute, so that there may be no conflict of interests. Will you not send to the Secretary the topics upon which you will write, so that the programme may be arranged at as early a day as possible?

C. H. Vilas, M. D., *President.* F. Park Lewis, M. D., *Secretary,*
188 Franklin Street, Buffalo, N. Y.

